# On the Roles of Wheat Endosperm ADP-Glucose Pyrophosphorylase Subunits

**DOI:** 10.3389/fpls.2018.01498

**Published:** 2018-10-16

**Authors:** Danisa M. L. Ferrero, Matias D. Asencion Diez, Misty L. Kuhn, Christine A. Falaschetti, Claudia V. Piattoni, Alberto A. Iglesias, Miguel A. Ballicora

**Affiliations:** ^1^Laboratorio de Enzimología Molecular, Instituto de Agrobiotecnología del Litoral (CONICET – UNL), Facultad de Bioquímica y Ciencias Biológicas, Universidad Nacional del Litoral, Santa Fe, Argentina; ^2^Department of Chemistry and Biochemistry, Loyola University Chicago, Chicago, IL, United States

**Keywords:** starch synthesis, allosteric regulation, 3-phosphoglycerate, heat stability, crop improvement, inorganic phosphate

## Abstract

The ADP-glucose pyrophosphorylase from wheat endosperm controls starch synthesis in seeds and has unique regulatory properties compared to others from this family. It comprises two types of subunits, but despite its importance little is known about their roles. Here, we synthesized *de novo* the wheat endosperm ADP-glucose pyrophosphorylase small (S) and large (L) subunit genes, heterologously expressed them in *Escherichia coli*, and kinetically characterized the recombinant proteins. To understand their distinct roles, we co-expressed them with well characterized subunits from the potato tuber enzyme to obtain hybrids with one S subunit from one source and an L subunit from the other. After kinetic analyses of these hybrids, we concluded that the unusual insensitivity to activation of the wheat endosperm enzyme is caused by a pre-activation of the L subunit. In addition, the heat stability and sensitivity to phosphate are given by the S subunit.

## Introduction

Nearly one-fifth of all calories consumed in the world come from wheat. In order to meet the caloric needs of our growing population, it is estimated that the yield of this crop must substantially increase in the near future (Altman, 1999). Crop yield is influenced by starch synthesis (Smidansky et al., 2003; Tuncel and Okita, 2013; Zi et al., 2018), which in higher plants and unicellular algae is controlled to a large extent by ADP-glucose (ADP-Glc) pyrophosphorylase (EC: 2.7.7.27; ADP-Glc PPase). This regulatory enzyme catalyzes the conversion of ATP and glucose-1-phosphate (Glc-1P) to form ADP-Glc and pyrophosphate (PP_i_) (Iglesias and Preiss, 1992; Ballicora et al., 2003, 2004). The wheat endosperm enzyme, like other plant ADP-Glc PPases, comprises two distinct homologous subunits, small (S) and large (L), which form an S_2_L_2_ heterotetramer. However, this endosperm form presents unique regulatory properties compared with enzymes from other sources. Despite the relevance of this particular enzyme, its impact on the agricultural importance of wheat, and its peculiar properties, little is known about their relationship to structure.

The majority of plant ADP-Glc PPases are allosterically activated by 3-phosphoglycerate (3-PGA) and inhibited by orthophosphate (P_i_) (Iglesias and Preiss, 1992; Ballicora et al., 2003, 2004). Although the main activator is 3-PGA, the enzymes may be slightly promiscuous toward low affinity secondary activators (Iglesias et al., 1993; Gomez-Casati and Iglesias, 2002; Kuhn et al., 2013). It has been established that dicot L subunits influence the different sensitivities to allosteric effectors based on their tissue localization (Kavakli et al., 2002; Crevillen et al., 2003). Since the divergence of monocots and dicots, dicot L subunits have duplicated and diversified into three separate branches of the phylogenetic tree, whereas monocot endosperm L subunits belong to a fourth distinct branch (Ballicora et al., 2004, 2005). It is known that monocot endosperm ADP-Glc PPases display unique responses to allosteric effectors (Plaxton and Preiss, 1987; Kleczkowski et al., 1993). For instance, the wheat (*Triticum aestivum*) enzyme purified from endosperm is insensitive to 3-PGA activation in absence of P_i_ (Gomez-Casati and Iglesias, 2002).

To reveal whether the unique regulatory properties of the wheat endosperm ADP-Glc PPase are determined by one subunit, or the combination of both, we constructed hybrid proteins mixing S and L subunits from different sources. We chose potato (*Solanum tuberosum*) tuber and wheat endosperm enzymes because of their distinct responses to allosteric effectors (Ballicora et al., 1998; Gomez-Casati and Iglesias, 2002). The potato tuber ADP-Glc PPase is highly sensitive to activation by 3-PGA, unlike the wheat endosperm enzyme. We analyzed the allosteric properties of the enzymes by combining the recombinant potato tuber S subunit (StuS) with either the potato tuber L subunit (StuL) or the wheat endosperm L subunit (TaeL) by heterologous expression in *Escherichia coli* of artificially synthesized genes. In addition, we studied the combinations of the wheat endosperm S subunit (TaeS) with either the wheat endosperm L subunit (TaeL) or the potato tuber L subunit (StuL).

## Materials and Methods

### Reagents

D-[^14^C]Glc-1P was purchased from GE Healthcare (Piscataway, NJ, United States). All other reagents were purchased at the highest quality available. The genes encoding the wheat endosperm ADP-Glc PPase small (TaeS) and large (TaeL) subunits were synthesized *de novo* (Bio Basic Inc., Markham, ON, Canada) (sequences in **Supplementary Figure 1**)

### Subcloning Procedures

TaeS and TaeL subunit genes, as well as genes encoding S and L subunits from potato tuber ADP-Glc PPase, were subcloned using *Nde*I and *Sac*I restriction sites in pET28c vectors, thus rendering proteins with an N-term His-tag after overexpression. In addition, all genes encoding the different S and L subunits were subcloned from the pET28c plasmid into the pCDFduet vector using *Nde*I and *Xho*I (downstream the *SacI* in the pET28 multi cloning sites). This construct overexpresses this construction overexpress the target protein but without any tag. By combining the pCDE and pET28 constructs, we obtained the different ADP-Glc PPases characterized in this work, with a His-tag on only one subunit. Although we obtained the entire set of combinations, we report here kinetic and regulatory data only from those heteromeric S/L enzymes where the His-tag was in the L subunit (using pET28c) and the S subunit was untagged (by using pCDFduet). All sequences were confirmed by the University of Chicago DNA Sequencing Facility (Chicago, IL, United States).

### Enzyme Expression and Purification

Proteins were over-expressed using *E. coli* BL21 (DE3) as a host. Cells were transformed with single pET28 or pCDFduet constructs for independent subunit production, or a combination of plasmids (pCDFduet/*small-subunit* with pET28c/*large-subunit*) as stated above. Transformed cells were grown in LB medium at 37°C until OD_600_ reached 0.6 and then cultures were induced with 0.5 mM IPTG for 16 h at 18°C. After induction, cells were harvested, resuspended in buffer A (50 mM MOPS pH 8.0, 5 mM MgCl_2_, 0.1 mM EDTA, 10% sucrose, 10 mM imidazole and 4 mM β-mercaptoethanol), disrupted by sonication and then centrifuged twice (10 min) at 30,000 ×*g.* Proteins were purified by immobilized metal ion affinity chromatography (IMAC) and all purification steps were performed at 4°C. Supernatants were loaded on a 1 ml His-Trap column (GE Healthcare) previously equilibrated with buffer A and proteins were eluted with a 10–300 mM imidazole linear gradient in buffer A (50 column volumes). Fractions containing the highest activity were pooled and concentrated. The resulting enzyme samples were >90% pure (according to densitometry of SDS-PAGE gels, not shown) and stored at −80°C until use. The recombinant wheat endosperm enzymes (TaeS/TaeL as well as single TaeS or TaeL subunits) remained fully active for 12 months.

### Molecular Mass Determination

Protein molecular mass was determined by gel filtration using a Tricorn 5/200 column (GE Healthcare) loaded with Superdex G200 resin (GE Healthcare). A Gel Filtration Calibration Kit-High Molecular Weight (GE Healthcare) with protein standards including thyroglobulin (669 kDa), ferritin (440 kDa), aldolase (158 kDa), conalbumin (75 kDa), and ovalbumin (44 kDa) was used. The column void volume was determined using a Dextran Blue loading solution (Promega, Fitchburg, WI, United States).

### Enzyme Activity Assays and Kinetic Analysis

A radiometric enzyme assay was employed to measure the synthesis of ADP-[^14^C]Glc from [^14^C]Glc-1P and ATP, as described before (Yep et al., 2004). The standard reaction mixture had 50 mM HEPPS (pH 8.0), 10 mM MgCl_2_, 4 mM dithiothreitol, 1.5 mM [^14^C]Glc-1P (100–1000 cpm/nmol), 2.5 mM ATP, 10 mM 3PGA (unless otherwise stated), 0.75 U/ml inorganic pyrophosphatase, 0.2 mg/ml bovine serum albumin, and enzyme in a total reaction volume of 0.2 ml. The reaction proceeded for 10 min at 37°C and was stopped in a boiling water bath for 1 min. The amount of ADP-[^14^C]Glc formed was quantified as described (Yep et al., 2004).

We also measured ADP-Glc PPase activity by analyzing ADP-Glc synthesis in a highly sensitive colorimetric method by following P_i_ formation after hydrolysis of PP_i_ by inorganic pyrophosphatase (Fusari et al., 2006). Reaction mixtures contained (unless otherwise specified) 50 mM MOPS pH 8.0, 10 mM MgCl_2_, 4 mM dithiothreitol, 2 mM ATP, 0.2 mg/ml bovine serum albumin, 0.5 U/ml yeast inorganic pyrophosphatase and a proper enzyme dilution. Assays were initiated by addition of 2 mM Glc-1P in a total volume of 50 μl. Reactions were incubated for 10 min at 37°C and terminated when the Malachite Green reagent was added. The complex formed with the released P_i_ was measured at 630 nm in an ELISA EMax detector (Molecular Devices).

One unit of activity (U) is defined as the amount of enzyme catalyzing the formation of 1 μmol of product per min, under conditions described above in each case.

Saturation curves were performed by assaying enzyme activity at different concentrations of the variable substrate or effector while the others remained constant. To avoid interference with any potential presence of S subunit homotetramers the saturation curves for 3-PGA were performed up to 1 mM. These homotetrameric S forms are not active until much higher concentrations of 3-PGA (Ballicora et al., 1995; Doan et al., 1999; Crevillen et al., 2003) or Fru-6P (unpublished). Experimental data were plotted as enzyme activity (U/mg) versus substrate (or effector) concentration (mM) and kinetic constants were determined by fitting the data to the Hill equation as described elsewhere (Crevillen et al., 2003; Kuhn et al., 2009). Fitting was performed with the Levenberg-Marquardt non-linear least-squares algorithm provided by the computer program Origin^TM^ 8.0. Hill plots were used to calculate the Hill coefficient (*n*_H_), the maximal velocity (*V*_max_), and the kinetic constants that correspond to the activator, substrate or inhibitor concentrations giving 50% of the maximal activation (*A*_0.5_), velocity (*S*_0.5_), or inhibition (*I*_0.5_). All kinetic constants are the mean of at least three independent sets of data, which were reproducible within a range of ± 10%.

### Protein Methods

Protein concentration was determined using the bicinchoninic acid reagent from Pierce Chemical Company (Rockford, IL, United States) as described previously (Smith et al., 1985). Samples were desalted using Bio-Rad 10 DG columns and concentrated with Centricon-30 devices (Amicon Inc., Billerica, MA, United States)). Recombinant proteins and purification fractions were defined by sodium dodecyl sulfate polyacrylamide gel electrophoresis (SDS-PAGE) according to Laemmli (Laemmli, 1970). Gels were loaded with 5–50 μg of protein per well and stained with Coomassie Brilliant Blue.

### Heat Stability

Heat stability of the recombinant constructs was measured as follows. Half milliliter of purified (by IMAC) TaeS/TaeL, StuS/TaeL, TaeS/StuL, StuS/StuL proteins were placed into individual 1.7 ml microcentrifuge tubes and centrifuged at 20,000 ×*g* for 15 min at 4°C. The enzyme samples were diluted to give the same final protein concentration (∼2 mg/ml). Then, 50 μl of the supernatant was aliquoted into five 1.7 ml microcentrifuge tubes and then placed at 0, 25, 37, 42, and 55°C for 5 min. Samples were immediately placed on ice for 2 min, centrifuged at 20,000 ×*g* for 15 min at 4°C and the supernatant was diluted with 50 mM MOPS buffer pH 8.0, 5 mM MgCl_2_, 0.1 mM EDTA, 0.5 mg/ml bovine serum albumin. The activity of 10 μl samples was assayed in duplicate as described above.

## Results

### Recombinant Wheat ADP-Glc PPase Expression and Characterization

To deepen the study of the regulatory properties of wheat endosperm ADP-Glc PPase, it was necessary to obtain a recombinant version of the enzyme. We artificially synthesized the respective genes coding for the S and L subunits with the codon usage for *E. coli* to maximize the heterologous expression in this bacterium. Our first attempts for kinetic analyses were based on a strategy where the subunits were obtained without a His-tag. The kinetic properties of the recombinant wild type wheat endosperm enzyme (untagged) were comparable to the native enzyme purified from wheat seeds, although this enzyme was unstable as previously described (Gomez-Casati and Iglesias, 2002). Then, we overexpressed the recombinant S and L subunits from wheat endosperm ADP-Glc PPase with an N-term His-tag, which allowed us to purify the proteins to high levels of purity (>90%), as shown in **Supplementary Figure 2A**. Interestingly, when we co-expressed the TaeS/TaeL heteromer with the His-tag in the L subunit, the enzyme became stable and remained active for several months. We confirmed that this stabilization effect could be ascribed to the N-term His-tag on the TaeL subunit, since, when we removed it the enzyme became unstable again (not shown). Thus, we decided to use this strategy (untagged S subunit; His-tagged L subunit) to produce the recombinant wheat endosperm ADP-Glc PPase and the hybrid S/L proteins characterized in this work. Gel filtration on Superdex-G200 revealed that in all the cases the purified recombinant enzymes had a quaternary structure of homo- (S_4_) or hetero-tetramers (S_2_L_2_) of about 200 kDa (see **Supplementary Figure 2B**). Also, the L subunits expressed alone were not active and thus not further characterized.

**FIGURE 1:**
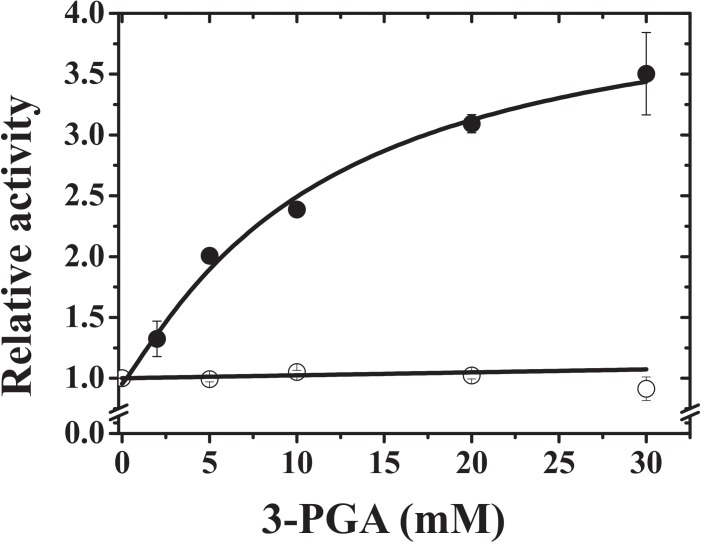
Saturation curves for 3-PGA of wheat ADP-Glc PPase. Black circles black circles belongs to homotetrameric TaeS enzyme, whilst in open circles belong to the heterotetrameric TaeL/TaeS. Relative activity was calculated considering activities of 0.04 and 4.7 U/mg for the homo- and heterotetrameric enzymes, respectively.

**FIGURE 2:**
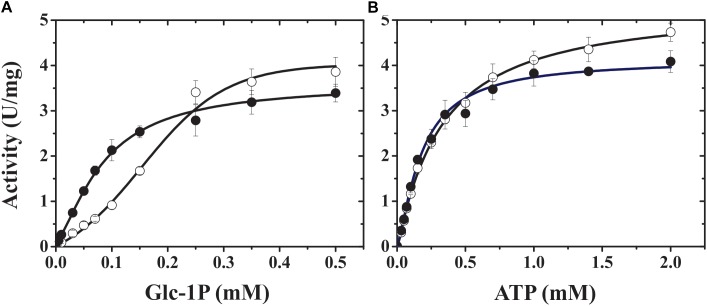
TaeL/TaeS saturation curves for both substrates, Glc-1P **(A)** and ATP **(B)**, in presence (dark circles) or absence (open circles) of 3-PGA.

The purified homotetrameric TaeS exhibited a relatively low specific activity (see **Supplementary Table 1**) and it was activated up to 3.5-fold by 3-PGA (**Figure 1** and **Supplementary Table 1**). Conversely, the heterotetrameric TaeS/TaeL showed insensitivity to 3-PGA (**Figure 1**). However, this metabolite increased by nearly twofold the apparent affinity of the enzyme toward Glc-1P and mainly affected the sigmoidal saturation curve for this substrate (**Figure 2**). The kinetic parameters of recombinant TaeS/TaeL detailed in **Table 1** are similar (in the same order of magnitude) to those previously reported for the ADP-Glc PPase purified from wheat seeds (Gomez-Casati and Iglesias, 2002). As a whole, these results indicate that the TaeL subunit significantly contributes to form a heterotetramer with relatively high catalytic capacity and also insensitivity to 3-PGA. These properties of the wheat endosperm ADP-Glc PPase agree with reports on the characterization of the enzyme from barley (Kleczkowski et al., 1993) and maize (Plaxton and Preiss, 1987) endosperm.

**Table 1:** Kinetic parameters for the recombinant TaeS/TaeL, TaeS/StuL, StuS/TaeL and StuS/StuL heteromeric ADP-Glc PPases.

Enzyme	Substrate	*S*_0.5_(mM)	*V*_max_(U.mg^−1^)	*n*_H_
TaeS/TaeL	Glc-1P	0.16 ± 0.01	4.1 ± 0.2	2.8
	Glc-1P + 3-PGA^∗^	0.083 ± 0.006	3.6 ± 0.2	1.3
	ATP	0.25 ± 0.01	4.7 ± 0.5	1.3
	ATP + 3-PGA^∗^	0.26 ± 0.01	4.7 ± 0.4	1.0
StuS/TaeL	Glc-1P	0.093 ± 0.004	36.4 ± 0.6	1.0
	Glc-1P + 3-PGA^∗^	0.072 ± 0.006	30.1 ± 0.8	1.0
	ATP	0.14 ± 0.01	48.4 ± 1.2	1.0
	ATP + 3-PGA^∗^	0.13 ± 0.02	33.6 ± 1.4	1.0
TaeS/StuL	Glc-1P	2.14 ± 0.18	0.050 ± 0.002	1.1
	Glc-1P + 3-PGA^∗^	3.4 ± 0.4	0.20 ± 0.01	1.0
	ATP	2.11 ± 0.34	0.030 ± 0.003	1.3
	ATP + 3-PGA^∗^	1.7 ± 0.2	0.43 ± 0.03	0.9
StuS/StuL	Glc-1P	2.4 ± 0.4	>1.9	1.4
	Glc-1P + 3-PGA^∗^	0.10 ± 0.02	18.5 ± 2.1	0.8
	ATP	1.6 ± 0.6	>0.8	1.2
	ATP + 3-PGA^∗^	0.06 ± 0.01	14.3 ± 0.4	1.2

*^∗^1 mM 3-PGA.*

### Kinetic Properties of Hybrid Constructs

**FIGURE 3:**
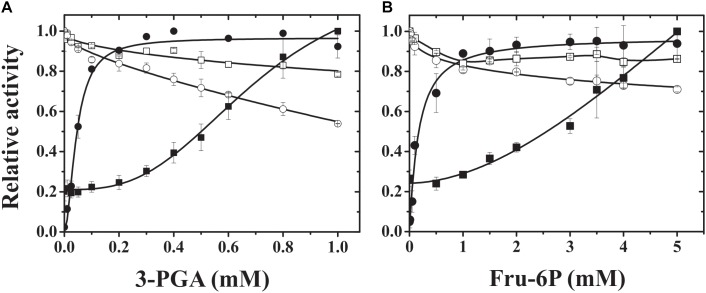
3-PGA **(A)** and Fru-6P **(B)** activation plots of hybrid ADP-Glc PPases. Saturation curves for 3-PGA and Fru-6P were performed for hybrid proteins TaeS/TaeL (open squares), StuS/TaeL (open circles), TaeS/StuL (dark squares), and StuS/StuL (dark circles). Relative activity was calculated considering the unity of activity as: 6.7, 56.8, 0.25, and 13.8 U/mg (in **A**) or 6.5, 55.2, 0.22, and 15.3 U/mg (in **B**), respectively for TaeS/TaeL, StuS/TaeL, TaeS/StuL, and StuS/StuL.

To further understand the properties of the different subunits from plant ADP-Glc PPases with different behavior toward effectors (such the potato tuber and wheat endosperm enzymes), we produced heteromeric S/L hybrid enzymes. For this purpose, we used the same strategy described above to obtain the recombinant wheat ADP-Glc PPase (see section Materials and Methods) by combining pCDFduet and pET28 constructs. We also expressed a recombinant potato tuber ADP-Glc PPase using this system, and the enzyme showed similar kinetic and regulatory properties to those previously reported (Iglesias et al., 1993; Ballicora et al., 1998, 1999, 2000). As shown in **Figure 3**, the presence of the respective L subunit from either potato tuber or wheat endosperm gave specific properties to the heteromeric hybrid proteins. Remarkably, the presence of StuL conferred sensitivity to 3-PGA and Fru-6P to the resulting hybrid enzymes, both of which are the main and a secondary activator of many ADP-Glc PPases from plants, respectively (Iglesias and Preiss, 1992; Iglesias et al., 1993; Gomez-Casati and Iglesias, 2002; Ballicora et al., 2003, 2004; Kuhn et al., 2013). Also, TaeS/StuL and StuS/StuL had a higher apparent affinity for 3-PGA than for Fru-6P (about five and threefold higher, respectively). For a more comparative analysis of the kinetic and regulatory properties of the hybrid ADP-Glc PPases, we determined their kinetic parameters in the absence or presence of 3-PGA. As shown in **Table 1**. TaeS/TaeL and StuS/TaeL enzymes were insensitive to activation, as 3-PGA had neither effect on *S*_0.5_ values for substrates nor on *V*_max_. Conversely, TaeS/StuL and StuS/StuL exhibited *V*_max_ values 9.7- and 42-fold higher (respectively) in presence of 3-PGA that in its absence.

Regarding their regulatory properties, the role of the S subunit from wheat endosperm ADP-Glc PPase seemed to be different from the L subunit. It appears that the S subunit may be primarily responsible for the sensitivity (in terms of relative apparent affinity) of the enzyme to P_i_ inhibition. For instance, constructs with StuS had a much higher affinity for P_i_ than those with TaeS (**Table 2**). StuS/StuL reached maximal inhibition of 4.6-fold and StuS/TaeL was inhibited 4.2-fold. TaeS/TaeL was inhibited 7.4-fold compared to 1.4-fold for TaeS/StuL, however, the apparent affinity for P_i_ of TaeS/TaeL was 40-, and 14-fold lower than StuS/StuL and StuS/TaeL, respectively (**Table 2**). The *I*_0.5_ for the TaeS/TaeL recombinant enzyme was in the millimolar-range, which is similar to the data from the enzyme purified from wheat seeds (Tuncel and Okita, 2013). In agreement with the idea that TaeS makes the enzyme less sensitive to P_i_ inhibition, the TaeS/StuL hybrid enzyme was unable to be inhibited more than 1.4-fold, even at high concentrations (10 mM) of P_i_.

**Table 2:** Pi inhibition of the proteins characterized in this work.

Enzyme	*I*_0.5_ (mM)	-Fold
StuS/StuL	0.17 ± 0.03	4.6
TaeS/StuL	n.d.^∗^	1.4
StuS/TaeL	0.50 ± 0.15	4.2
TaeS/TaeL	6.8 ± 0.3	7.4

*n.d.^∗^, not determined, as low inhibition level was observed even at 10 mM P_i_*.

### Heat Stability

**FIGURE 4:**
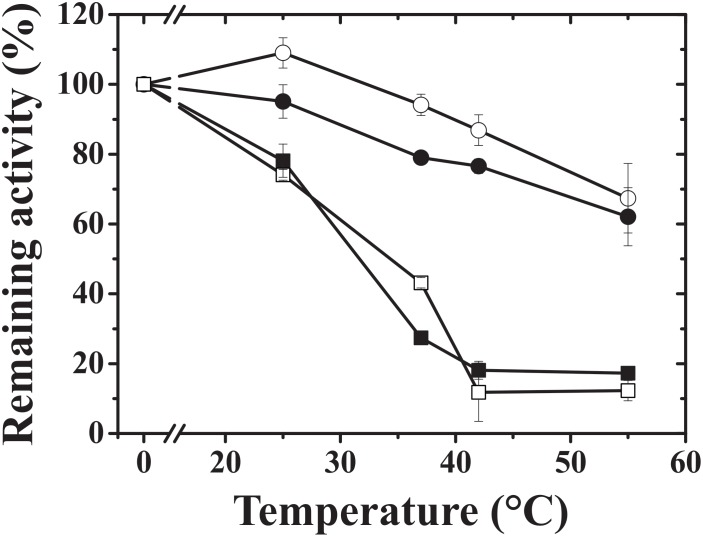
Heat stability of hybrid ADP-Glc PPases. Assays were carried out as described in section Materials and Methods; TaeS/TaeL (open squares), StuS/TaeL (open circles), TaeS/StuL (dark squares), and StuS/StuL (dark circles).

We noticed that the hybrid proteins were more stable and retained more activity than the wheat endosperm wild-type protein (TaeS/TaeL). As a result, we tested the heat stability of the hybrid constructs to determine the cause of this effect. The hybrids exhibited similar heat stability for 5 min at 37°C, but at 55°C, StuS/TaeL retained almost 80% of the original activity, whereas TaeS/StuL retained no more than 10% (**Figure 4**). TaeS/TaeL could not retain the original activity above 42°C. StuS/StuL was previously shown to remain active until 60°C (Yep et al., 2004). The presence of the StuS subunit seems to be responsible for the increased heat-stability of the StuS/TaeL hybrid construct.

## Discussion

Herein, we report the recombinant production of the wheat endosperm ADP-Glc PPase by a procedure that allowed us to reach a high level of purity and stability of the enzyme. The pure recombinant enzyme exhibited kinetic and regulatory proteins that agree with those previously reported for the enzyme purified from its natural source (Gomez-Casati and Iglesias, 2002). Also, the recombinant procedure was useful to construct chimeric hybrid proteins by combining small and large subunits between the wheat endosperm and the potato tuber heterotetrameric ADP-Glc PPases. Because these latter enzymes have distinctive properties with respect to allosteric regulation and sensitivity to heat treatments (Iglesias et al., 1993; Ballicora et al., 1995, 2003, 2004; Gomez-Casati and Iglesias, 2002), we could explore specific roles played by the small and large subunits in the regulation and structural stability of plant ADP-Glc PPase.

Our results support the view that the large subunit in plant ADP-Glc PPase plays a main role in conferring regulatory properties to the enzyme. The TaeL subunit was found inactive, in agreement with homologous proteins from other plants (Kavakli et al., 2002; Ballicora et al., 2003, 2004; Crevillen et al., 2003). However, TaeL showed responsibility for a major contribution to the “pre-activation” and insensitivity to activators (either 3-PGA or Fru-6P) for StuS/TaeL and TaeS/TaeL. This is compatible with the idea that L subunits influence the regulatory properties of ADP-Glc PPases. Previous data from hybrid potato-*Arabidopsis* ADP-Glc PPases showed that the apparent affinity for 3-PGA was mainly dependent upon the L subunit regardless of the S subunit involved (Ventriglia et al., 2007). In the case of the wheat endosperm enzyme, the TaeL causes pre-activation/insensitivity to the activator rather than altering the apparent affinity for it.

Concerning the small subunit of plant ADP-Glc PPase, results herein suggest a main role of the protein in conferring apparent affinity to P_i_ inhibition; the subunit is also critical for the heat stability of the enzyme. Previous studies have shown the heat stability of ADP-Glc PPases may influence yield or seed weight in rice, maize, wheat, and potato tuber (Krauss and Marschner, 1984; Wilhelm et al., 1999; Smidansky et al., 2002; Morita et al., 2005). In early controlled studies, heat adversely affected the seed biomass of wheat (Gibson and Paulsen, 1999) and the rate of photosynthesis (Nagai and Makino, 2009). Many ADP-Glc PPases are heat-stable up to 60°C, whereas the enzymes from endosperm are heat labile (Preiss et al., 1971; Kleczkowski et al., 1993; Ballicora et al., 1998; Gomez-Casati and Iglesias, 2002). The heat stability of the potato tuber ADP-Glc PPase enzyme has been attributed to the presence of Cys^12^ in StuS, which forms a disulfide bridge in the homotetrameric protein (Ballicora et al., 1999; Jin et al., 2005). This residue is conserved in all S subunits from plants, with the exception of endosperm enzymes from monocots (Ballicora et al., 1999). Previously, the heat stability of the maize endosperm was improved by introducing a mutation from methionine to cysteine at the N terminus of the S subunit (Linebarger et al., 2005).

One way to increase yield in a warmer climate may be to engineer a heat stable ADP-Glc PPase (Giroux et al., 1996). In our study, the StuS/TaeL enzyme exhibited increased heat stability and similar allosteric properties to the TaeS/TaeL wild-type enzyme. We propose that this hybrid construct may yield a heat stable transgenic wheat plant, without major changes in physiology. Previously, a transgenic wheat plant was constructed using a genetically modified maize ADP-Glc PPase L subunit and a mutant potato-maize mosaic S subunit (Meyer et al., 2007). They saw an increased yield, but only in an environment with non-limiting resources. Unlike their work, the potato/wheat hybrid we propose contains unaltered S and L subunit genes from two agriculturally important crops.

## Conclusion

Regarding enzymes from storage tissues (e.g., seeds, tubers, etc.), it seems that one main difference in regulation between monocots and dicots is their sensitivity and response to activators. Dicot ADP-Glc PPases are very sensitive to allosteric effectors and can be activated to a high *V*_max_, whereas some monocot forms are in a seemingly insensitive state. In this work we show that the insensitivity in wheat is caused by the L subunit, which drives the heterotetramer to a more active form when compared to the S subunit alone. This unifies the theory that the interaction between L and S subunits of plant ADP-Glc PPases determines their different regulatory properties for adaptation to the needs of different tissues (Crevillen et al., 2003). In this case, wheat endosperm is not an exception, but rather an extreme case of how the L subunit affects the heteromer regulatory properties. Rather than improving the affinity of 3-PGA, it activates the heterotetramer and renders it insensitive for further activation.

## Author Contributions

MK, MA, DF, AI, and MB conceived and designed the experiments and wrote the paper. MK, CF, and DF performed the experiments. MK, MF, CP, MA, AI, and MB analyzed the data. AI and MB contributed reagents, materials, and analysis tools.

## Conflict of Interest Statement

The authors declare that the research was conducted in the absence of any commercial or financial relationships that could be construed as a potential conflict of interest.

## Abbreviations

3-PGA: 3-phosphoglycerate
ADP-Glc: ADP-glucose
ADP-Glc PPase: ADP-glucose pyrophosphorylase
Fru-6P: fructose-6-phosphate
P_i_: inorganic orthophosphate
PP_i_: inorganic pyrophosphate
StuL: *Solanum tuberosum* tuber L subunit
StuS: *Solanum tuberosum* tuber S subunit
TaeL: *Triticum aestivum* endosperm L subunit
TaeS: *Triticum aestivum* endosperm S subunit
