# Supplementary material for: On the Roles of Wheat Endosperm ADP-Glucose Pyrophosphorylase Subunits
Source: Front Plant Sci. 2018 Oct 16;9:1498. doi: 10.3389/fpls.2018.01498 (PMC6232684; doi:10.3389/fpls.2018.01498)
Supplement: Supplementary file 1 [file Data_Sheet_1.PDF]

Supplemental Table 1. Kinetic parameters for the homotetrameric TaeS ADP-Glc PPase

Substrate	$S_{0.5}$ (mM)	V_{max} (U/mg)	n_H
Glc-1P	> 15[a]	> 0.14 ± 0.01 (at 15 mM Glc-1P)	N/A[c]
Glc-1P + 3PGA[b]	> 15[a]	> 0.18 ± 0.01 (at 15 mM Glc-1P)	N/A[c]
ATP	2.6 ± 0.2	0.099 ± 0.005	1.1
ATP + 3-PGA[b]	2.2 ± 0.2	0.27 ± 0.05	1.7

[a] Saturation curves for Glc-1P did not reach saturation when assayed up to 20 mM

[b] Concentration of3-PGA was 5 mM

[c] Not applicable
