# Supplementary material for: On the Roles of Wheat Endosperm ADP-Glucose Pyrophosphorylase Subunits
Source: Front Plant Sci. 2018 Oct 16;9:1498. doi: 10.3389/fpls.2018.01498 (PMC6232684; doi:10.3389/fpls.2018.01498)

<u>Supplementary Figure 2:</u> Determination of the structure of the different ADP-Glc PPases versions. **(A)** Reducing SDS-PAGE and **(B)** determination of molecular mass by gel filtration chromatography of recombinant proteins: TaeL/StuS (open square), TaeL/TaeS (open circle), StuL/StuS (dark circle) and StuL/TaeS (dark square). M: molecular mass marker.

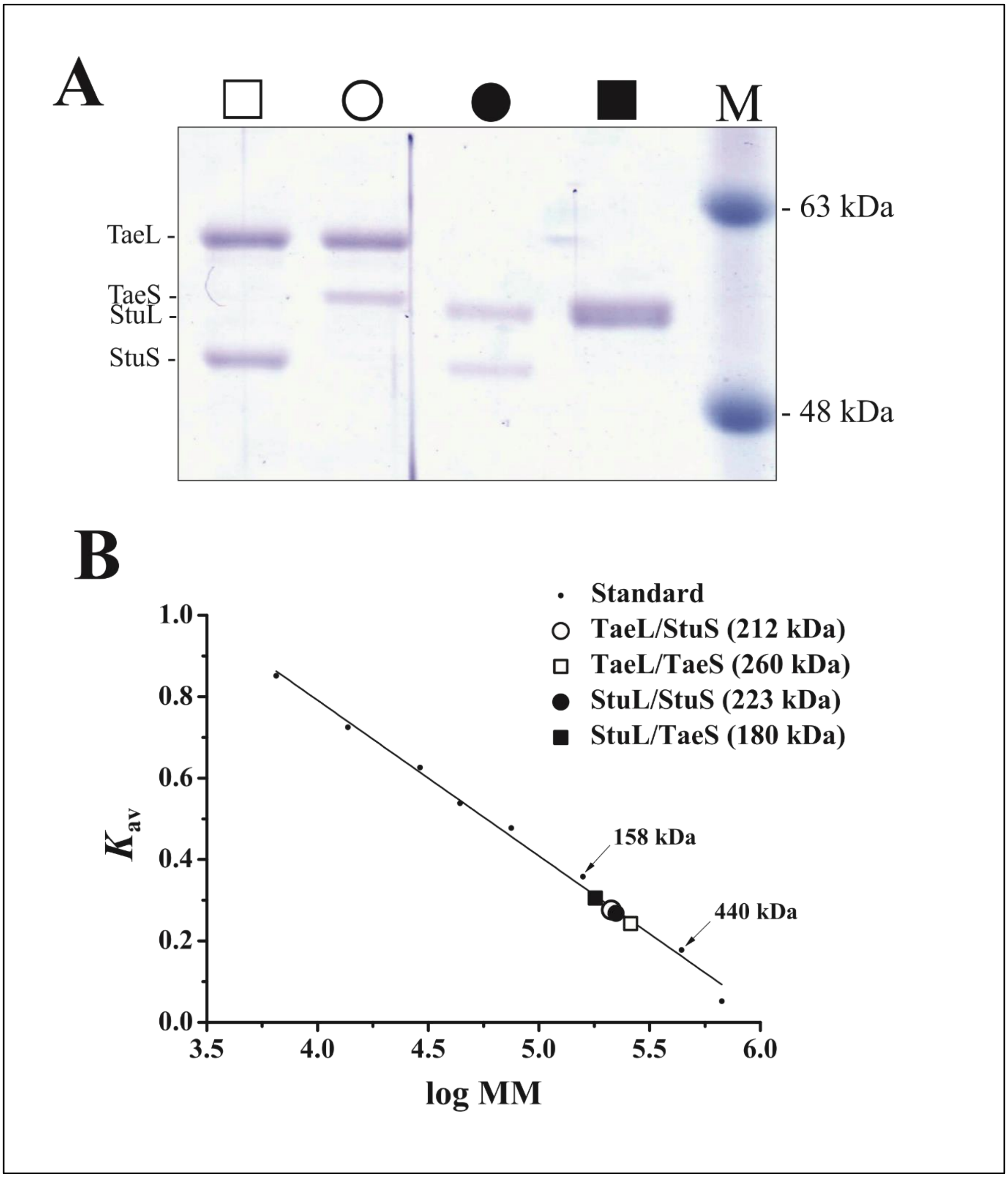

Supplement: Supplementary file 2 [file Data_Sheet_2.pdf]
