# Supplementary material for: On the Roles of Wheat Endosperm ADP-Glucose Pyrophosphorylase Subunits
Source: Front Plant Sci. 2018 Oct 16;9:1498. doi: 10.3389/fpls.2018.01498 (PMC6232684; doi:10.3389/fpls.2018.01498)
Supplement: Supplementary file 3 [file Image_1.PDF]

```
                10         20         30         40         50
        ....|....| ....|....| ....|....| ....|....| ....|....|
TaeES   ---------- ---------- ---------- ---------- -------MDV
TaeEL   MSSMQFSSVL PLEGKACISP VRREGSASER LKVGDSSSIR HERASRRMCN
                60         70         80         90        100
        ....|....| ....|....| ....|....| ....|....| ....|....|
TaeES   PLASKTFPSP SPSKREQCNV DGHKSSSKHA DLNPHANDSV LGIILGGGAG
TaeEL   GGRGPAATGA QCVLTSDASP ADTLVLRTSF RRNYADPNEV AAVILGGGTG
               110        120        130        140        150
        ....|....| ....|....| ....|....| ....|....| ....|....|
TaeES   TRLYPLTKKR AKPAVPLGAN YRLIDIPVSN CLNSNISKIY VRTQFNSASL
TaeEL   TQLFPLTSTR ATPAVPIGGC YRLIDIPMSN CFNSGINKIF VMTQFNSASL
               160        170        180        190        200
        ....|....| ....|....| ....|....| ....|....| ....|....|
TaeES   NRHLSRAYGS NIGGYKNEGF VEVLAAQQSP -DNPDWFQGT ADAVRQYLWL
TaeEL   NRHIHRTY-L GGGINFTDGS VEVLAATQMP GEAAGWFRGT ADAVRKFIWV
               210        220        230        240        250
        ....|....| ....|....| ....|....| ....|....| ....|....|
TaeES   FEEH---NVM E-YLILAGDH LYRMDYEKFI QAHRETDADI TVAALPMDEE
TaeEL   LEDYYKNKSI EHILILSGDQ LYRMDYMELV QKHVDDNADI TLSCAPVGES
               260        270        280        290        300
        ....|....| ....|....| ....|....| ....|....| ....|....|
TaeES   RATAFGLMKI DEEGRIIEFA EKPKGEQLKA MMVDTTILGL DDARAKEMPY
TaeEL   RASEYGLVKF DSSGRVVQFS EKPKGDDLEA MKVDTSFLNF AIDDPAKYPY
               310        320        330        340        350
        ....|....| ....|....| ....|....| ....|....| ....|....|
TaeES   IASMGIYVIS KHVMLQLLRE QFPGANDFGS EVIPGATSTG MRVQAYLYDG
TaeEL   IASMGVYVFK RDVLLNLLKS RYAELHDFGS EILPRALHDH N-VQAYVFTD
               360        370        380        390        400
        ....|....| ....|....| ....|....| ....|....| ....|....|
TaeES   YWEDIGTIEA FYNANLGITK KPIPDFSFYD RSAPIYTQPR HLPPSKVLDA
TaeEL   YWEDIGTIRS FFDANMALCE QP-PKFEFYD PKTPFFTSPR YLPPTKSDKC
               410        420        430        440        450
        ....|....| ....|....| ....|....| ....|....| ....|....|
TaeES   DVTDSVIGEG CVIKNCKIHH SVVGLRSCIS EGAIIEDTLL MGADYYETEA
TaeEL   RIKEAIISHG CFLRECKIEH SIIGVRSRLN SGSELKNAMM MGADSYETED
               460        470        480        490        500
        ....|....| ....|....| ....|....| ....|....| ....|....|
TaeES   DKKLLAEKGG IPIGIGKNSH IKRAIIDKNA RIGDNVMIIN VDNVQEAARE
TaeEL   EISRLMSEGK VPIGVGENTK ISNCIIDMNA RIGRDVVISN KEGVQEADRP
               510        520
        ....|....| ....|....| ....|
TaeES   TDGYFIKSGI VTVIKDALLP SGTVI
TaeEL   EEGYYIRSGI VVIQKNATIK DGTVV
```

Supplementary Fig. 1 Amino acid sequences of the wheat endosperm S (TaeS) and L (TaeL) subunit ADP-Glc PPases.
